# Anatomy of the neural fibers at the superior mesenteric artery—a cadaver study

**DOI:** 10.1007/s00423-022-02529-1

**Published:** 2022-05-03

**Authors:** Michael D. Reinehr, Raphael N. Vuille-dit-Bille, Christopher Soll, Anubhav Mittal, Jaswinder S. Samra, Ralph F. Staerkle

**Affiliations:** 1 Institute of Pathology and Molecular Pathology, University Hospital Zurich, Zurich, Switzerland; 2 Department of Pediatric Surgery, University Children’s Hospital of Basel, Basel, Switzerland; 3 ventravis–Practice for Abdominal Surgery, Cham, Switzerland; 4 University of Zurich, Zurich, Switzerland; 5 Hirslanden Klinik St. Anna, St. Anna-Strasse 32, 6006 Lucerne, Switzerland; 6 Upper Gastrointestinal Surgical Unit, Royal North Shore Hospital, Sydney, Australia; 7 Australian Pancreatic Center, Sydney, Australia; 8 University of Notre Dame of Australia, Fremantle, Australia; 9 University of Lucerne, Lucerne, Switzerland

**Keywords:** Neural fibers, Anatomy, Superior mesenteric artery, Pancreatic surgery, Cadaver

## Abstract

**Purpose:**

Most surgeons perform right-sided semicircular clearance of the superior mesenteric artery (SMA) nerve plexus for pancreatic head carcinoma, presuming a linear course of the SMA nerve fibers. The hypothesis was that the SMA nerve plexus fibers follow a non-linear course, and the goal of the present study was to assess the neural fibers distribution along the SMA.

**Methods:**

The course of neural fibers along the retropancreatic and suprapancreatic SMA was assessed in 7 cadavers.

**Results:**

In the retropancreatic course of the vessel, the main nerve cords branch and form a large number of finer nerve branches performing an anti-clockwise rotation of slightly less than 90° around the SMA. Finer nerve branches are located rather close to the vessel, while the main nerve cords are localized in the loose connective tissue of the peripheral parts of the vascular sheath. Nerve fibers around the suprapancreatic SMA run as two main nerve cords framing the artery on the right lateral-ventral and the left lateral to lateral-dorsal side.

**Conclusion:**

The rotation of the nerve fiber around the SMA indicates that a more radical resection of at least 180° of neural tissue around the SMA might be required to achieve tumor clearance in pancreatic cancer with perineural invasion at the uncinate margin.

## Introduction

Pancreatic ductal adenocarcinoma (PDAC) is one of the most aggressive malignancies and the seventh leading cause of cancer-related mortality globally [1]. Incidence continues to increase in both sexes [2]. Due to lack of specific symptoms during the early stages of the disease, diagnosis is often made at a more advanced stage, and only 15 to 20% of patients have a resectable tumor at time of diagnosis [3, 4]. Depending on tumor stage, surgery with or without neoadjuvant chemotherapy represents the standard therapy for resectable tumors [5]. Median survival of patients with resectable or borderline resectable PDAC undergoing upfront resection is about 20 months with a 5-year survival rate of 17% [1, 6, 7]. It has been hypothesized that one of the reasons for the poor long-term survival is that PDAC spreads early along the peripancreatic neural plexus and that this spread can lead to positive resection margins [8, 9]. Positive resection margins have been reported in as many as 40% of resected patients and this correlates with significant reduction in the overall survival [10].

Dissection of the retroperitoneal nerve plexus, especially around the superior mesenteric artery (SMA), has hence been proposed by some authors to improve the quality of the surgical resection [11–13]. Nevertheless, current evidence does not show a survival benefit [3]. Furthermore, complete resection of the retroperitoneal nerve plexus can lead to severe diarrhea and malnutrition [3]. However, data in the literature indicating severity and intractability of the diarrhea are missing [14]. Therefore, it is imperative that the course of neural fibers along the SMA (i.e., spiral vs. longitudinal vs. random pattern) is documented to allow for bespoke surgery and reduction in the morbidity associated with complete resection of the retroperitoneal nerve plexus.

The aim of this study is to describe the distribution of neural fibers around the SMA so a designer clearance can be performed based on the orientation of the neural fibers abutting the tumor.

## Materials and methods

### Ethics

Cadavers from the body donation program of the Institute for Anatomy of the University of Zurich, Switzerland, were used. The body donation program follows the guidelines of the Swiss Academy of Medical Sciences (SAMW). All donors had filled out an informed consent before they died. The study was approved by the local ethics committee (KEK-ZH Nr. 2015–0381).

### Inclusion–exclusion criteria

Cadavers ≥ 18 years old at the time of death were included. Cadavers with either trauma or surgery to tumor at the region of interest were excluded.

### Tissue preparation

All specimens were collected at the Institute for Anatomy of the University of Zurich, Switzerland, and analyses were performed at the Institute of Pathology and Molecular Pathology, University Hospital Zurich, Switzerland. Cadavers were fixed using the Thiel-embalming technique [15, 16] or a common formalin fixation technique with 4% formaldehyde as described elsewhere [15]. Tissue specimens including pancreas, aorta, inferior vena cava, SMA, and superior mesenteric vein were removed en bloc.

Pancreatic specimens from these cadavers were subsequently trimmed into tissue blocks measuring 2 × 3 cm (frontal plane) surrounding the centrally enclosed SMA. Edges of the tissue blocks were marked using ink (from Cancer Diagnostics, Inc., Durham, NC, USA) in different colors (green — right side; blue — left side; red — cranial side; black — caudal side) maintaining the original orientation of the tissue blocks within the cadaver to enable later 3-dimensional orientation of histological slides. Specimens were then cut into blocks of about 4 mm thickness in the frontal plane and histologically processed as described elsewhere [17]. Briefly, tissues were fixed in 4% paraformaldehyde and paraffin embedded. Tissue shrinkage of up to 20% occurs artificially when the tissue is processed into paraffin blocks [18–20]. All tissue blocks were completely worked up in steps of 200 μm thickness, and cut into 2–3 μm slides being stained with hematoxylin/eosin. In addition, slides were stained immunohistochemically using a polyclonal rabbit anti-human S100 antibody (Z0311 from Agilent, Santa Clara, CA, USA), in order to show the exact course of nerve fibers. Therefore, staining was performed on a Ventana Benchmark Ultra System with an OptiViewDAB Kit (Ventana Medical Systems, Inc., Oro Valley, AZ, USA) using CC1 (Ventana Medical Systems Inc.) as a pretreatment step (8 min incubation time) for antigen retrieval. The tissue sections were incubated with the primary antibody for 32 min (dilution 1:2000).

For virtual microscopy and archiving, slides were digitalized using a Nano Zoomer C9600 Virtual Slide Light microscope scanner using NDP, View Software, version 2.7.25 (Hamamatsu Photonics Germany GmbH, Herrsching am Ammersee, Germany). Exported images then were analyzed using Photoshop CS6 Extended (Version 13.0 × 64, Adobe Systems Inc., San José, CA, USA) and PowerPoint 2016 (Microsoft, Redmond, WA, USA).

### Macroscopic preparation

In a formalin-fixed specimen consisting of aortic cuff with SMA and complete adherent pancreas, the pancreatic tissue on both sides of the SMA was removed, and the remaining pancreatic and connective tissue surrounding the SMA was successively removed with a scalpel and splinter forceps, sparing the nerve fibers (anatomic preparation).

### Analysis of nerve fiber course around the SMA

To analyze the course of the fibers of the nerve plexus surrounding the SMA, the complete tissue blocks with the pancreatic tissue surrounding the SMA were histologically processed perpendicular to the course of the SMA in steps of 200 μm. The individual slides were oriented and aligned at the same magnification using the ink markings and then arranged in the correct order to reconstruct the entire tissue block. This resulted in a sequence of 120 to 165 individual slides per specimen.

## Results

### Cadavers

Cadavers from 7 donors (3 males and 4 females) were included in the present study. The median age of the cadavers was 78 years (range 69 to 81 years). Microscopic analysis was carried out in 6 cadavers. Additionally, a macroscopic preparation was performed in one specimen.

### Nerve fiber course around the SMA

#### Suprapancreatic portion of the SMA

**Fig. 1:**
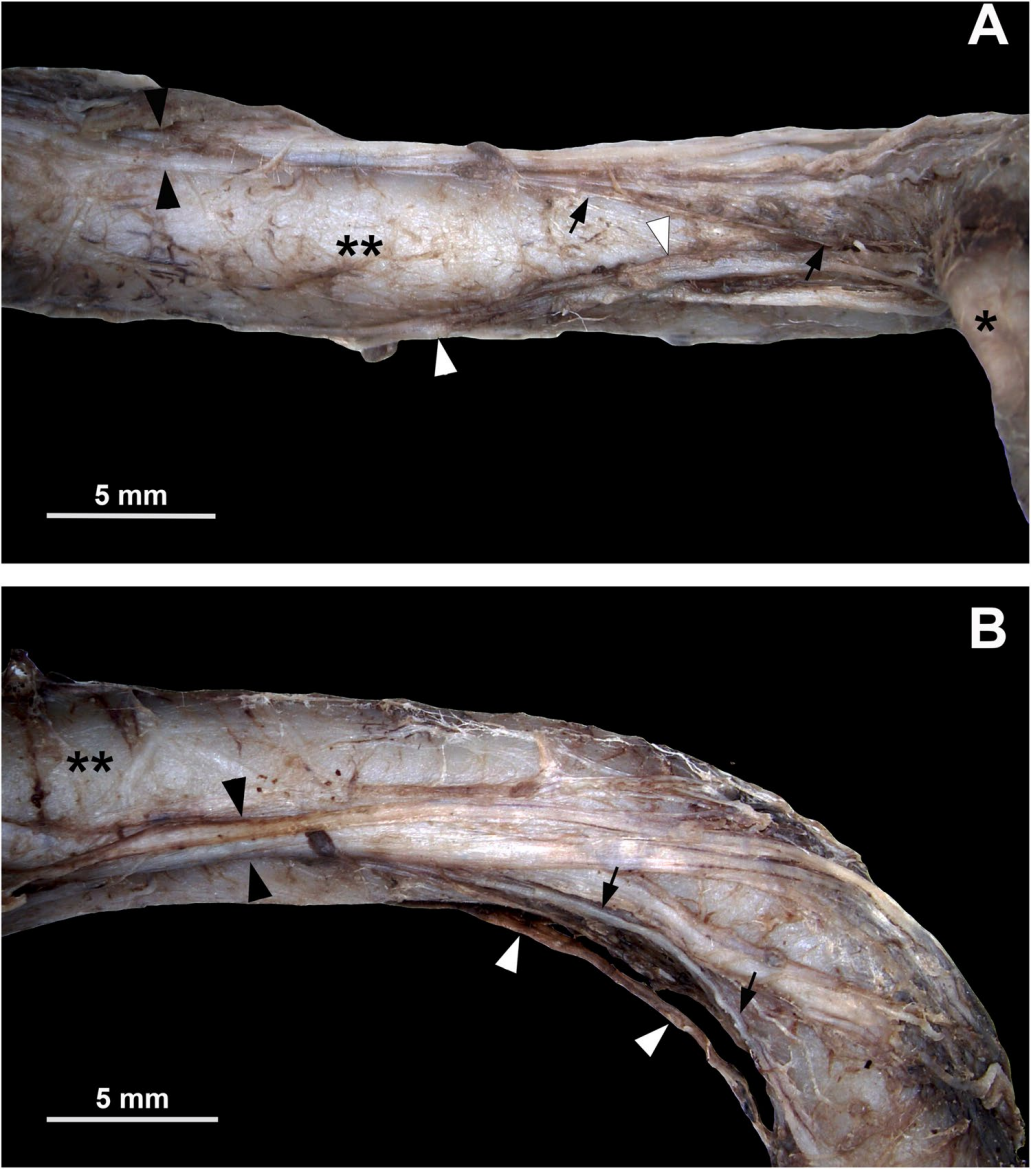
Macroscopy of SMA and nerve plexus. Shown is the SMA (double asterisk) from ventral (**A**) and from left lateral (**B**). Surrounding fat and connective tissue have been removed. In the proximal part of the artery (left border in the pictures), the two circumscribed major nerve cords are visible on the left lateral-dorsal side (black arrow heads) and on the right lateral-ventral side (white arrow heads) of the artery, showing a slightly less than 90° spiral (counterclockwise) rotation along their course. Both major nerve cords branch into smaller fibers (black arrows mark the branching fibers of the left latero-dorsal nerve cord) to the branching IPDA (single asterisk)

**Fig. 2:**
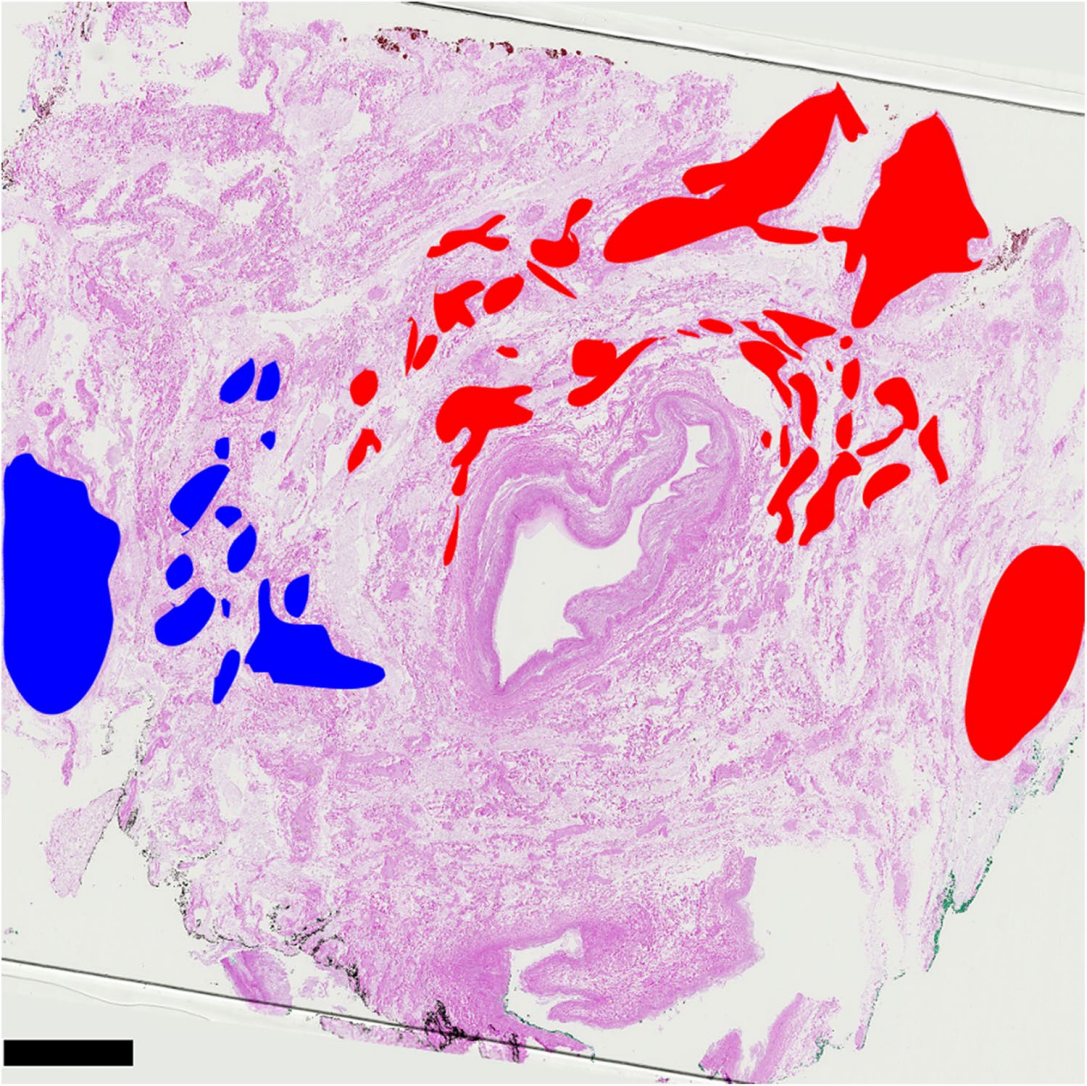
Nerve plexus surrounding the proximal SMA. A representative histological section (hematoxylin and eosin staining) through the proximal (pre-pancreatic) part of the SMA with its surrounding nerve plexus is shown. Larger nerve fiber cords are visible, which accompany the SMA as two main strands in the loose connective tissue of the peripheral parts of the vascular sheath: right lateral-ventral (marked red) and left lateral-dorsal (marked blue). The smaller branches located rather close to the vessel showing a small overlap in the border area of both main strands, but no extensive intertwining can be seen. Scale bar: 5 mm

**Fig. 3:**
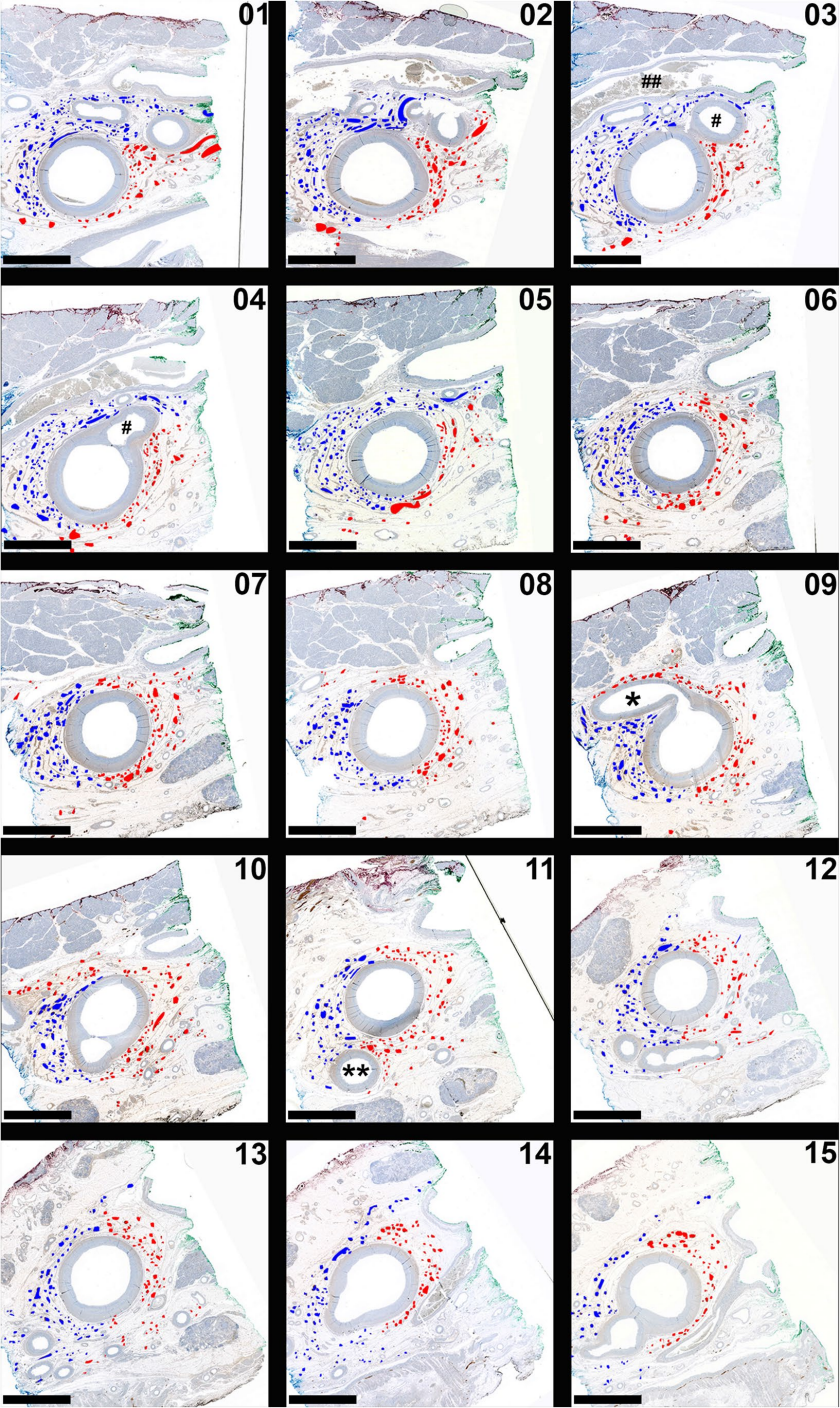
Retropancreatic course of the superior mesenteric plexus. Exemplary histological specimens (S100 immunohistochemical staining) taken over the entire retropancreatic course of the SMA (approximately 2.8 cm) from proximal (**01**) to distal (**15**) are shown; (**01**) located about 4 mm distal to the section shown in Fig. 3. Branches originating from the right lateral-ventral (marked red) and from the left lateral-dorsal (marked blue) plexus show a slight (slightly less then 90°) spiral (anti-clockwise) course along the distal SMA, showing a minimal overlap in the marginal areas but no significant intertwining. Fibers originating from the left lateral-dorsal (blue) branch into smaller fibers dorsal to the IPDA, and the right lateral-ventral (red) plexus branches into smaller fibers ventral to the IPDA (asterisk in **09**). An accessory inferior pancreatic artery (hashtag in **03**) as first branch of the SMA is seen, which is located directly adjacent to the splenic vein (double hashtag in **03**) dividing into a right and a left branch (**01** and **02**). The middle colic artery is marked with double asterisks (**11**). Scale bars: 5 mm

In all specimens analyzed, the part of the SMA superior to the pancreas showed an arrangement in two thicker main nerve cords framing the artery on the right lateral-ventral and the left lateral to lateral-dorsal side. These two main cords rotate approximately 60° anti-clockwise (looking towards distal SMA) per centimeter around the SMA (Figs. 1 and 2). Here, numerous finer nerve branches could be identified that were located rather close to the vessel, while the main trunks were rather localized in the loose connective tissue of the peripheral parts of the vascular sheath (Fig. 3). Especially the finer branches showed only slight overlaps in the peripheral parts of the two strands.

#### Retropancreatic portion of the SMA

**Fig. 4:**
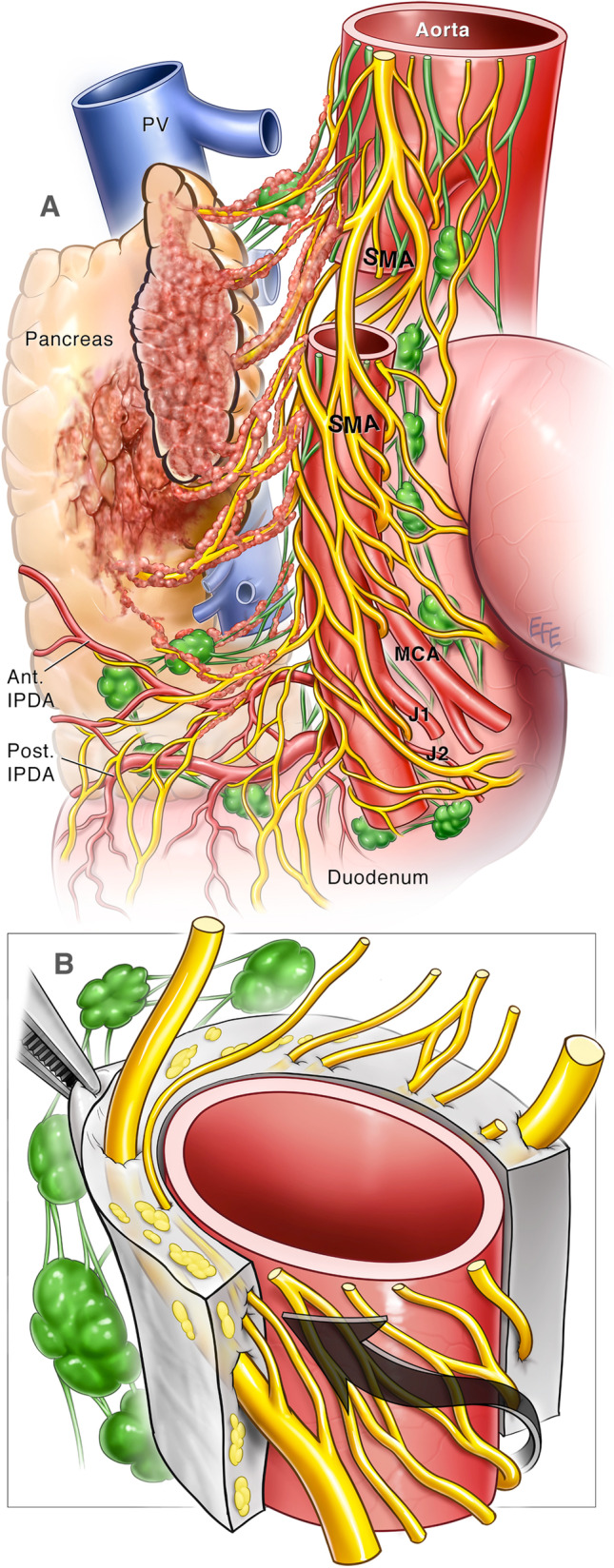
Course of the superior mesenteric plexus. PV, portal vein; IPDA, inferior pancreaticoduodenal artery; SMA, superior mesenteric artery; MCA, middle colic artery; J1, first jejunal branch of the SMA; J2, second jejunal branch of the SMA. **A** Relationship between the tumor, the vessels, and the mesenteric plexus. Tumor spread along the neural fibers. **B** Relationship between the SMA, the neural fibers, and the lymphatic vessels

In retropancreatic portion of the SMA, the main strands branched very quickly and formed a large number of finer nerve branches which accompanied the SMA and showed an anti-clockwise rotation of slightly less than 90° around the vessel in their part accompanied by pancreatic tissue (Figs. 2 and 4). Thinning of the nerve fibers was observed over the entire length of the SMA in all 6 samples examined, but the thin fibers always remained relatively evenly distributed around the circumference of the vessel. Furthermore, they continued to show only a discrete overlap in the peripheral areas of the nerve fiber bundles emanating from the two main cords.

#### Branching of the inferior pancreaticoduodenal artery

Branches originating from the left latero-dorsal (red) and from the right latero-ventral (blue) main nerve cords (Fig. 2 and 4) continue to accompany the branching inferior pancreaticoduodenal artery (IPDA) with the latero-dorsal fibers building the anterior (red) and the latero-ventral fibers building the dorsal (blue) nerve plexus around the IPDA.

## Discussion

To our knowledge, this is the first time that the retropancreatic distribution of the neural fibers along the SMA is described in detail.

In 1958, Yoshioka and Wakabayashi first descripted the concept of the nerve plexus around the pancreatic head [21]. The Japan Pancreas Society recently published the seventh edition of the General Rules for the Study of Pancreatic Cancer. The General Rules underline the importance of perineural invasion in patients with PDAC [22]. In accordance with the publication of Yoshioka and Wakabayashi, seven categories for neural plexuses are described by the Japan Pancreas Society: pancreatic head plexus I (PL ph I), pancreatic head plexus II (PL ph II), superior mesenteric arterial plexus (PL sma), common hepatic artery plexus (PL cha), plexus within hepatoduodenal ligament (PL hdl), splenic plexus (PL sp), and celiac plexus (PL ce). In particular, the invasion of PL ph I and PL sma was found to have a correlation with patient prognosis and lymph node involvement along the SMA [23].

Imamura et al. recently published a study to determine the impact of tumor abutment to the branches of the SMA on survival [24]. They showed in multivariate analysis that abutment to the SMA branches is an independent predictor of poor overall survival after surgery (*P* = 0.001). Also, regarding the initial recurrence pattern, abutment to the SMA branches was significantly associated with high incidence of distant metastasis (*P* < 0.001). It seems that tumor abutment of the SMA branches represents an advanced local stage of PDAC. In the present study, we could show that at least the IPDA receives neural fibers from the right latero-ventral and the left latero-dorsal main nerve cords. Therefore, one could argue that if a branch of the SMA shows tumor abutment, a more radical clearance of the perineural tissue around the SMA is needed.

In 2007, the concept of the mesopancreas was introduced by Gockel et al. [8]. Yi and colleagues published recently a study analyzing the relationships among the mesopancreas and pancreatic head plexus from the morphological, developmental, and clinical perspectives [25]. It was believed that incomplete resection of the mesopancreas is responsible for the high rate of R1 resections after pancreatic head resections. Yi et al. showed that there is a complex relationship between the PL sma, the pancreatic head/uncinate process, and the aortocaval plane. Basically, tumor can spread in different directions in this region. These findings would again advocate for a more aggressive clearance of this region during pancreaticoduodenectomy.

The main findings of the current cadaver study showed that there are two main nerve cords framing the SMA on the right lateral-ventral and the left lateral to lateral-dorsal side. These two main nerve cords show an anti-clockwise rotation of almost 90° during the peripancreatic course of the SMA and just a slight overlap of the finer nerve fibers. Therefore, since the fibers from both cords rotate 90°, a clearance of 180° is required to remove potentially affected neural tissue. In a study by Ohigashi et al. in patients with KRAS mutated pancreatic cancer in which the distribution of this mutation on the right vs. the left side of the PL sma was investigated, there was a lack of detection on the left side facing away of the tumor [26]. This can be explained by our finding that there is no diffuse interweaving of the nerve fibers but only a slight helical course. Taken together with the findings from Yi and Imamura, this would suggest that a more radical resection of at least 180° of neural tissue around the SMA is required to achieve tumor clearance in tumors in the pancreatic head/uncinate process [24, 25].

The present study is limited by its low sample size (*n* = 7 cadavers). Hence, factors possibly affecting the anatomy of the neural fibers at the superior mesenteric artery (including the tumor itself) were not assessed. Nevertheless, findings (i.e., fiber course around the SMA) were very robust among the included cadavers. Furthermore, the present study did not assess if a more radical resection affects postoperative outcomes (including diarrhea and malabsorption) or not.

## Conclusion

The high prevalence of neural invasion in pancreatic cancer and its strong association with local tumor recurrence after curative tumor resection mandates a wider excision of the neural tissue around the SMA of at least 180° based on the current cadaveric study. The present study indicates that future research is required to investigate if a more circumferential resection of neural tissue around the SMA affects patients’ outcomes. Furthermore, the hereby observed pattern of neural fibers at the superior mesenteric artery should be assessed in patients with pancreatic head carcinoma.
